# Response: Commentary: Elimination of Left-Right Reciprocal Coupling in the Adult Lamprey Spinal Cord Abolishes the Generation of Locomotor Activity

**DOI:** 10.3389/fncir.2018.00062

**Published:** 2018-08-02

**Authors:** Andrew D. McClellan

**Affiliations:** ^1^Division of Biological Sciences, University of Missouri, Columbia, MO, United States; ^2^Interdisciplinary Neuroscience Program, University of Missouri, Columbia, MO, United States

**Keywords:** locomotion, central pattern generators, oscillators, coordination, coupling

In the CNS, central pattern generators (CPGs) produce the basic temporal pattern of electrical motor activity that produces rhythmic behaviors such as locomotion. A major issue in motor control is whether CPGs are organized into distinct modules that can function autonomously or if the different CPG modules are interdependent (Figure 1 in Messina et al., 2017). Regarding the previous commentary (Cangiano and Grillner, 2018), there are at least two relevant questions concerning the locomotor rhythm generating capabilities of CPG modules in right and left hemi-spinal cords in adult lampreys: (a) Does the burst activity generated by hemi-spinal cords represent well-coordinated locomotor activity? (b) Does generation of burst activity by hemi-spinal cords represent a normal physiological capability of these spinal circuits, or is this capability manifested under certain, possibly artificial, experiment conditions? In the text below, all page numbers and figures refer to those in our previous study (Messina et al., 2017).

For our previous study, a longitudinal midline (ML) lesion was performed along the rostral spinal cord of adult lampreys, and rostral muscle activity (adjacent to ML spinal lesion) and caudal muscle activity (below ML spinal lesion) were recorded in response to sensory stimulation of the anterior head (Figure 3A). The rationale was that initiation of spinal motor activity in whole animals in response to sensory stimulation and descending brainstem activation would provide the most physiological situation possible in which to test the rhythm generating capabilities of hemi-spinal cords (also see Jackson et al., 2005). First, under these conditions, sensory stimulation elicited coordinated rostral and caudal locomotor muscle burst activity (Figure 3B1), but with somewhat higher than normal burst proportions (BP) and intersegmental rostrocaudal phase lags (Φ_INT_) (Figure 3C). Second, following a subsequent spinal transection at the caudal end of the ML lesion in the same animals (Figure 3A), thereby *isolating* rostral right and left hemi-spinal cords from intact caudal cord, sensory stimulation usually elicited tonic, unpatterned rostral muscle activity (Figure 3B2). However, for ~30% of the trials, stimulation could elicit relatively high frequency rostral ipsilateral “burstlet” activity (Figures 5–7, 9). Importantly, there are at least six features of “burstlet” activity suggesting that it does not represent well-coordinated locomotor activity (p. 14). Our muscle “burstlet” activity (Messina et al., 2017) and the *in vitro* “high-frequency rhythm” that is assumed to represent locomotor activity (Cangiano and Grillner, 2003, 2005) have some significant differences, and it is uncertain if they are equivalent.

In the previous commentary (Cangiano and Grillner, 2018), there were several incomplete, misleading, or incorrect statements. First, the commentary stated that stimulation of the *in vitro* lamprey hemi-spinal cord initiates a “bout of locomotor burst activity” consisting of “well-coordinated bursting.” However, the term “well-coordinated locomotor activity” embodies more than just burst frequency, but also should include data for BP and Φ_INT_ (Wallén and Williams, 1984; Boyd and McClellan, 2002). Because BP and Φ_INT_ are obvious and easy parameters to measure, it is surprising that these parameters were not reported in the previous studies (Cangiano and Grillner, 2003, 2005). For the “burstlet” activity in our study, plots of BP vs. cycle time (CT) usually were abnormal (Figures 6A2–C2), and phase lags usually were absent (Figure 9; p. 9, lower right column).

Second, the previous commentary stated that *prior* to a more caudal spinal transection in our study, the muscle burst activity produced by rostral hemi-spinal cords was “driven from the intact [caudal] part of the spinal cord.” However, in our experiments under these conditions, average Φ_INT_ values were positive (Figures 3B1, 3C3) and did not change significantly with CT (unpublished), suggesting that intact caudal spinal circuits were not driving (activating) bursts in the rostral hemi-spinal cords. Also, experimental evidence does not support this proposed activation mechanism in the lamprey spinal cord. Lastly, BP and Φ_INT_ values for rostral muscle burst activity (adjacent to spinal ML lesion) were only moderately higher than those during normal swimming (Figures 3C2, 8; p. 9), suggesting that the CPGs in rostral hemi-spinal cords were functional when connected to intact caudal spinal locomotor circuitry.

Third, the previous commentary stated that in our study, the presence of rostral muscle burst activity *prior* to a more caudal spinal transection (Figures 3A, 3B1) was not a convincing test of the integrity of the CPGs in rostral hemi-spinal cords. They suggested that the mini-scalpel blade we used to make ML spinal lesions might have damaged the spinal cord, and they showed a misleading image of the “dorsal view” of this blade (i.e., wider, non-cutting edge). (a) The spinal cords of animals in our study were ~200–300 μm thick (~1 mm wide), only the very tip of this blade was used for cutting, and right and left halves of the spinal cord often separated slightly during ML lesioning. (b) For ~35% of the experiments in Cangiano and Grillner (2003), ML spinal lesions were made with a 0.4 mm dia. needle, which is more blunt and wider than the tip of the blade we used, yet in their previous study there were no reported experimental differences using the needle compared to using an ophthalmic blade.

Fourth, the previous commentary stated that the frequencies of muscle “burstlet” activity for our experiments (Messina et al., 2017) were “somewhat higher” than those obtained for their *in vitro* experiments (Cangiano and Grillner, 2003, 2005). However, for our experiments, the average (~25 Hz) and maximum (>60 Hz) “burstlet” frequencies were about 4–5 times higher than those observed for their *in vitro* experiments (~6 and ~12–15 Hz, respectively). (Note: the somewhat higher burst frequencies in Cangiano et al., 2012 presumably occurred because recordings were made within minutes after performing ML lesions, when the excitability of hemi-spinal cords probably was very high.) Also, the average and maximum “burstlet” frequencies in our study were ~6 and ~10 times higher than the average and maximum muscle burst frequencies, respectively, during swimming for normal whole animals (McClellan et al., 2016). The relatively high “burstlet” frequencies appear to be due to changes in the properties of hemi-spinal cords, such as an increase in excitability because of the lack of left-right coupling (p. 13) and possibly lesion-induced cellular and synaptic plasticity (Hoffman and Parker, 2010).

Fifth, the previous commentary stated that the “isolated condition [*in vitro* spinal cord] is a much cleaner situation” than in our whole-animal experiments. However, we are not convinced that synchronous, non-specific, high-frequency (33 Hz) stimulation of the surface of hyper-excitable *in vitro* hemi-spinal cords (Cangiano and Grillner, 2003, 2005) is cleaner or more physiological compared to sensory stimulation and descending brainstem activation of hemi-spinal cords in whole animals (Messina et al., 2017; also see Jackson et al., 2005).

Sixth, the previous commentary incorrectly stated that in our paper we claimed “that the burst generation is crucially dependent on reciprocal inhibition.” On the contrary, we stated that “reciprocal inhibition mainly regulates left-right phasing of [lamprey] locomotor activity and is not critical for rhythmogenesis” (p. 14; also see Hagevik and McClellan, 1994). In addition, we emphasized that right-left coupling involves both reciprocal inhibition as well as reciprocal excitation (p. 14, 15; Figure 1). Thus, blocking left-right reciprocal inhibition is not a definitive test for rhythmogenesis of hemi-spinal cords because right and left motor networks would still be coupled by reciprocal excitation.

Seventh, the previous commentary stated that the higher than normal BP values for “burstlet” activity in our study (Figures 6A2–C2) are expected, thereby implying that the “burstlet” activity represents lamprey locomotor activity. Indeed, the higher than normal BPs are expected (p. 13), but this represents a small part of only 1 of the 6 reasons we provided to suggest that our “burstlet” activity does not represent well-coordinated swimming activity (p. 14). For example, for the majority of our experiments, BP values for “burstlet” activity changed significantly with CT (Figures 6A2–C2), which is not characteristic of swimming motor activity in the lamprey.

In conclusion, our experiments employed the most physiological conditions possible for testing the rhythm generating capabilities of hemi-spinal cords in adult lampreys. Our results strongly suggest that isolated right and left spinal cord modules are not autonomous and, by themselves, do not generate coordinated ipsilateral locomotor burst activity.

## Author contributions

The author confirms being the sole contributor of this work and approved it for publication.

### Conflict of interest statement

The author declares that the research was conducted in the absence of any commercial or financial relationships that could be construed as a potential conflict of interest.

## References

[B1] Boyd M. McClellan A. D. (2002). Variations in locomotor activity parameters versus cycle time in larval lamprey. *J. Exp*. Biol. 23, 3707–3716. 10.1242/jeb.205.23.3707 12409497

[B2] Cangiano L. Grillner S. (2003). Fast and slow locomotor burst generation in the hemispinal cord of the lamprey. J. Neurophysiol. 89, 2931–2942. 10.1152/jn.01100.2002 12611971

[B3] Cangiano L. Grillner S. (2005). Mechanisms of rhythm generation in a spinal locomotor network deprived of crossed connections: the lamprey hemicord. J. Neurosci. 25, 923–935. 10.1523/JNEUROSCI.2301-04.2005 15673673 PMC6725629

[B4] Cangiano L. Grillner S. (2018). Commentary: elimination of left-right reciprocal coupling in the adult lamprey spinal cord abolishes the generation of locomotor activity. Front. Neural Circuits 12:34 10.3389/fncir.2018.00034 29893384 PMC5990913

[B5] Cangiano L. Hill R. H. Grillner S. (2012). The hemisegmental locomotor network revisited. Neuroscience 210, 33–37. 10.1016/j.neuroscience.2012.03.007 22433298

[B6] Hagevik A. McClellan A. D. (1994). Coupling of spinal locomotor networks in larval lamprey revealed by receptor blockers for inhibitory amino acids: neurophysiology and computer modeling. J. Neurophysiol. 72, 1810–1829. 10.1152/jn.1994.72.4.1810 7823103

[B7] Hoffman N. Parker D. (2010). Lesioning alters functional properties in isolated spinal cord hemisemental networks. Neuroscience 168, 732–743. 10.1016/j.neuroscience.2010.04.014 20394805

[B8] Jackson A. W. Horinek D. Boyd M. McClellan A. D. (2005). Disruption of left-right reciprocal coupling in the spinal cord of larval lamprey abolishes brain-initiated locomotor activity. J. Neurophysiol. 94, 2031–2044. 10.1152/jn.00039.2005 16000521

[B9] McClellan A. D. Pale T. Messina J. A. Buso S. Shebib A. (2016). Similarities and differences for swimming in larval and adult lampreys. Physiol. Biochem. Zool. 89, 294–312. 10.1086/686893 27327180

[B10] Messina J. A. St Paul A. Hargis S. Thompson W. E. McClellan A. D. (2017). Elimination of left-right reciprocal coupling in the adult lamprey spinal cord abolishes the generation of locomotor activity. Front. Neural Circuits 11:89 10.3389/fncir.2017.00089 29225569 PMC5705556

[B11] Wallén P. Williams T. L. (1984). Fictive locomotion in the lamprey spinal cord *in vitro* compared with swimming in the intact and spinal lamprey. J. Physiol. 347, 225–239. 10.1113/jphysiol.1984.sp015063 6142945 PMC1199444

